# Supplementary material for: Chromothripsis during telomere crisis is independent of NHEJ, and consistent with a replicative origin
Source: Genome Res. 2019 May;29(5):737–49. doi: 10.1101/gr.240705.118 (PMC6499312; doi:10.1101/gr.240705.118)
Supplement: Supplemental Material [file supp_gr.240705.118_Supplemental_file_1.zip › contigs/annotated_contigs/DB93/contig.3.DB93_length_710_mean_cov_8.87323943662.docx]

**DB93_length_710_mean_cov_8.87323943662**

```
CCAGCTAATTTTTTGTATTTTAGGTAGAGACGGGGTTTCACTGGGTTAGCCAGGATGGTCTC|TG|TACCTTCAGATACCCAGTTGGCC
 >chr3:128403571-128403633 - E=2e-25                             >chr6:12083322-1208373
TTCTTCATAGACATATGAGTGTATATGTCTGTCAAGTGTGTGTGTGTGTTTCTGTACATTTCTACACATTCATCTTCATGGCGCAAAAT
0 + E=1e-230 p=0e+00
GTTTAGAATTATGTCTTTTTAATATAATATATGTTGAGCATTTTCCATCAATAAATATTTTGTAAAAAATACATTTAATGGCTAAACAA

CATTCTCTTGTATAGAAATATTTAAGTATTTGTTTAACCAGCTCCATATTGTTTTGTATATAGAATTCCTGCCAAGTTATTATTAACAA

AAATGCTGGAGTAATATCGCAATAGCAATTCATTTATTAGACAACTGTGTGTCAAGGGTCTAGTGACCCTGGCAACTATGTGCTCTGTG

GAGTAAGATACAGTTTGATGTGGAAAGCA|TCATTTTACCATTTATA|TTTTCATTTTACCATTTATATTTTCTCCAATTACTAAAGTA
                                               >chr6:12083085-12083306 - E=4e-121
CCTATAATGAATACATATCTTAATATAAACTGTGTAATTTAAATCCAATAAAAGCTAACAATGTGCTCAATTATCTTTTAAAAAATGTT

GGCTATAGATTCCAAATTGCGAAGTTCCAATCATACAGAAAACTACAGAATAATTCCTAAAACATGGAAACATTTACACACATAGTCCA

TT
```
